# Supplementary material for: Climate Dynamics: A Network-Based Approach for the Analysis of Global Precipitation
Source: PLoS One. 2013 Aug 19;8(8):e71129. doi: 10.1371/journal.pone.0071129 (PMC3747276; doi:10.1371/journal.pone.0071129)
Supplement: Text S1 — Shortest path of significant nodes of the network. (PDF) [file pone.0071129.s001.pdf]

## Shortest path of significant nodes of the network

The shortest path, $d_{ij}$, of three nodes in Australia, Africa and US is reported in Figures S1, S2, and S3, respectively. The Australia and Africa nodes lie in the supernode set of the network, while the US node has intermediate values of both the degree centrality and the weighted average topological distance.

The Australia node (coordinates: 141.25° E, 26.25° S, Figure S1) belongs to the supernode region of Eastern Australia and has a remarkably strong connection with the whole Australian continent. From a topological point of view, short distance regions are South Africa and the Indonesian Archipelago. Quite well-connected regions are also located in the Mongolian area, Northern and Central America. An animated version of the shortest path of the present node is visible in Movie S1.

The Africa node (coordinates: 3.75° W, 13.75° N, Figure S2) is part of the supernode area located in the Sahel region, which also presents the lowest weighted average topological distance values of the network. Apart from the Central Africa zone, which is directly connected to the present node, there is a clear connection with many regions such as Central Asia, India, Southern Europe, Northern Africa, Eastern Australia, southern part of South America and US. An animated version of the shortest path of the Africa node is visible in Movie S2.

The US node (coordinates: 105° W, 41.25° N, Figure S3) is quite intensely linked to the whole North America continent, which also defines its physical neighborhood. Much weaker connections are found with the Atlantic Coast of South America, Eastern Australia and the Sahel region. An animated version of the shortest path of the US node is shown in Movie S3.

Movies S4, S5, S6, and S7 are animations of the shortest path maps illustrated in panels A, B, C, and D of Fig. 7 (main text), respectively.
